# Pre-Diagnostic Plasma 25-Hydroxyvitamin D Levels and Risk of Non-Melanoma Skin Cancer in Women

**DOI:** 10.1371/journal.pone.0035211

**Published:** 2012-04-06

**Authors:** Geyu Liang, Hongmei Nan, Abrar A. Qureshi, Jiali Han

**Affiliations:** 1 Key Laboratory of Environmental Medicine Engineering of Ministry of Education, School of Public Health, Southeast University, Nanjing, China; 2 Channing Laboratory, Department of Medicine, Brigham and Women’s Hospital and Harvard Medical School, Boston, Massachusetts, United States of America; 3 Department of Dermatology, Brigham and Women’s Hospital, Harvard Medical School, Boston, Massachusetts, United States of America; 4 Department of Epidemiology, Harvard School of Public Health, Boston, Massachusetts, United States of America; 5 Division of Cancer Epidemiology, Department of Epidemiology and Public Health, University of Maryland School of Medicine, Baltimore, Maryland, United States of America; Brigham & Women's Hospital, and Harvard Medical School, United States of America

## Abstract

**Background:**

Recent reports have shown that vitamin D status was inversely associated with the risk of various cancers. However, few studies examined the association between vitamin D levels and risk of skin cancer.

**Methods:**

We prospectively evaluated the association between baseline plasma 25(OH)D levels and the risk of incident squamous cell carcinoma (SCC) and basal cell carcinoma (BCC) among 4,641 women from the Nurses’ Health Study (NHS) and the NHS II with 510 incident BCC cases and 75 incident SCC cases. We used multivariate logistic regression models to calculate odds ratios (ORs) and 95% confidence intervals (CIs).

**Results:**

Plasma 25(OH)D levels were positively associated with risk of BCC after adjusting for age at blood draw, season of blood draw, lab batch, hair color, burning tendency, the number of sunburns, and ultra-violet B flux of residence at blood collection. Women in the highest quartile of 25(OH)D had more than 2-fold increased risk of BCC compared with women in the lowest quartile (OR = 2.07, 95% CI = 1.52–2.80, P for trend <0.0001). We also found a significantly positive association between plasma 25(OH)D levels and SCC risk after adjusting for the same covariates (OR, highest vs. lowest quartile  = 3.77, 95% CI = 1.70–8.36, P for trend  = 0.0002).

**Conclusion:**

In this prospective study of women, plasma vitamin D levels were positively associated with non-melanoma skin cancer risk. Considering that most circulating vitamin D is due to sun exposure, the positive association between plasma vitamin D and non-melanoma skin cancer is confounded by sun exposure. Our data suggest that one-time measurement of plasma vitamin D levels may reasonably reflect long-term sun exposure and predict the risk of non-melanoma skin cancer.

## Introduction

Skin cancer, the most common malignancy, has been increasing rapidly over the past decades in the United States, especially in women [1]–[3]. Mounting epidemiologic evidence has suggested that vitamin D may be associated with reduced risk of various types of cancers, including colorectal [4], prostate [5], breast [6], pancreatic [7], and lung cancers [8]. However, few studies have examined the association between vitamin D levels and risk of skin cancer, and the data are inconsistent [9]–[11].

It is more difficult to study the relationship between vitamin D and skin cancer than other internal cancers because vitamin D is predominantly produced in the skin by ultraviolet B (UVB) exposure, which is the well-established risk factor for skin cancer. UVB exposure-synthesized vitamin D in the skin usually contributes 80–90% to total vitamin D in the human body [12]. Only small amount of vitamin D is derived from other sources including dietary intake. 25-hydroxyvitamin D [25(OH)D] is produced in the liver by a hydroxylation reaction [13]. Circulating plasma 25(OH)D is considered to be the best biomarker of vitamin D status because it reflects the total vitamin D levels [14]. Three epidemiologic investigations have suggested conflicting associations of skin cancer with vitamin D levels [9]–[11]. In an analysis of the Osteoporotic Fractures in Men Study, inverse association between plasma vitamin D levels and risk of non-melanoma skin cancer in elderly men was found [9]. However, in two other recent analyses of health maintenance organization populations, higher plasma vitamin D levels were significantly associated with increased risk of non-melanoma skin cancer [10], [11]. Although experimental studies have shown that vitamin D treatment can inhibit proliferation of melanoma and basal cell carcinomas *in vitro* [15], [16], recent evidence in a large cohort of postmenopausal women suggests that daily supplementation of vitamin D did not reduce incidence of skin cancer [17]. However, this study using low dose vitamin D supplementation (400 IU/day) may not offer informative evidence regarding the potential influence of vitamin D status on skin cancer development [17].

Here we prospectively assess the association between plasma 25(OH)D levels and the risk of non-melanoma skin cancer in a nested case-control study among women from two large ongoing cohort studies: the Nurses’ Health Study (NHS) and NHS II.

## Methods

### Ethics Statement

The institutional review board of Brigham and Women’s Hospital approved this study. Participants’ completion and return of the self-administered questionnaire was considered to imply informed consent.

**Table 1:** Characteristics by quartile of plasma vitamin D concentrations in NHS and NHS II.

25-Hydroxyvitamin D concentrations, ng/mL									
	NHS					NHS II			
	1st quartile	2nd quartile	3rd quartile	4th quartile		1st quartile	2nd quartile	3rd quartile	4th quartile
n	484	518	535	558		629	629	639	649
Age at blood draw (y)	57.5(7.1)	57.6(6.7)	57.9(6.9)	58.0(6.8)		44.6(4.2)	44.0(4.2)	43.6(4.4)	43.4(4.4)
Red or blonde hair color (%)	16.7	16.1	15.9	18.7		19.3	20.1	23.6	20.3
Burning tendency (%)	35.6	38.9	38.4	32.2		33.0	25.7	24.1	24.1
Numbers of sunburns (≥5, %)	5.7	6.3	8.8	6.3		8.8	8.8	10.2	10.1
Season of blood draw									
Summer (%)	20.7	30.9	30.1	36.6		10.1	15.5	22.3	28.8
Spring and Fall (%)	45.3	48.3	43.9	44.4		57.4	57.9	57.6	56.5
Winter (%)	34.1	20.9	26.0	19.0		32.5	26.6	20.1	14.8
UVB flux (>113, %)	26.1	28.6	38.4	37.4		41.3	41.8	48.5	50.5

Values are presented as means (Standard deviation) or percentages.

### NHS Nested Case-control Study

In 1976, 121,700 female registered nurses between 30 and 55 years old were enrolled in the NHS. Women completed an initial questionnaire and have been followed biennially by questionnaire to update information on exposure status and to identify newly diagnosed case subjects of cancer and other medical conditions. Between 1989 and 1990, blood samples were collected from 32,826 participants for analysis. Measurements of plasma 25(OH)D levels were available from a subset of the women who served as controls in several nested case-control studies of chronic diseases conducted previously, including breast cancer, colon polyps, colon cancer and ovarian cancer [18]–[21]. Eligible cases in this study were Caucasian women with incident skin squamous cell carcinoma (SCC) or basal cell carcinoma (BCC) occurring after blood collection but before 2008. The rest of the subjects were controls. Participants who had previously diagnosed skin cancer before blood collection were excluded from the analysis. The nested case-control study consisted of 387 BCC cases, 67 SCC cases, and 1,641 controls.

### NHS II Nested Case-control Study

The NHS II was established in 1989 among 116,609 female registered nurses between the ages of 25 and 42. Participants completed biennial mailed questionnaires to update exposure status and disease diagnoses. Between 1996 and 1999, 29,611 participants provided blood samples. We used all the controls from previous nested case-control studies of chronic disease within the NHS II blood cohort that had been analyzed for vitamin D, including hypertension, breast cancer and ovarian cancer [19], [22], [23]. The inclusive and exclusive conditions of cases and controls were the same as for the NHS. Eligible cases were Caucasian women with incident skin SCC or BCC occurring after blood collection and the rest of the subjects were controls. The follow-up ended in 2007. Participants who had previously diagnosed skin cancer before blood collection were excluded. The nested case-control study consisted of 123 BCC cases, 8 SCC cases, and 2,415 controls.

### Identification of BCC and SCC

We have routinely identified cases of BCC and SCC in both cohorts. Participants reported new diagnoses biennially. With their permission, participants’ medical records were obtained and reviewed by physicians to confirm their self-reported diagnosis. Only pathologically confirmed invasive SCC cases were included in this study. Medical records were not obtained for self-reported cases of BCC, but the validity of BCC self-reports was more than 90% in validation studies in our cohorts in early years [24], [25]. In this analysis, cases were diagnosed after blood collection and up to 2008 (NHS) and 2007 (NHS II).

### Measurement of Plasma 25(OH)D

Plasma 25(OH)D concentrations were measured using radioimmunoassay or chemiluminescence immunoassay, which have been described in detail previously [26], [27]. In the NHS, laboratory assays were completed in 14 batches of 4 studies from 1996 to 2004 (3 batches in 1996, 4 batches in 2000, and 7 batches in 2004). In the NHS II, blood samples were assayed in 3 batches of 3 studies (1 batch in 2003 and 2 batches in 2005). Blinded replicate quality-control samples were interspersed throughout the sets for assessing variability. The intra-assay coefficients of variation (CV) were <17.6%.

### Statistical Analysis

In the nested case-control study of SCC or BCC, 25(OH)D measurements (continuous in ng/mL) were categorized into quartiles according to the distribution in control population of NHS and NHS II study separately. Odds ratios (ORs) and 95% confidence intervals (95% CIs) were calculated to examine the relationship between plasma 25(OH)D levels and SCC or BCC by multivariate logistic regression models. Age at blood draw (in years), season of blood draw [summer (June-August), fall (September-November), winter (December-February), and spring (March-May)], and laboratory batch were included as independent variables in all models. Hair color (1 = red, 2 = blonde, 3 = light brown, 4 = dark brown, 5 = black), burning tendency (1 = practically none, 2 = some redness only, 3 = burn, 4 = painful burn, 5 = painful burn with blisters), the number of sunburns (1 = never, 2 = 1–2, 3 = 3–5, 4 = ≥6), and average annual UV-B flux at residence (≤113 and >113) were also adjusted for in the multivariate models. Pooled analyses of two cohort studies were conducted by merging data sets.

**Table 2:** Odds ratios of BCC by quartile of plasma vitamin D concentrations in NHS and NHS II.

	25-Hydroxyvitamin D concentrations, ng/mL				P for trend
	1st quartile	2nd quartile	3rd quartile	4th quartile	
**NHS**					
Quartile values	≤20.4	20.4–27.0	27.0–34.2	>34.2	
n, case/control	69/406	86/420	108/405	124/410	
OR^a^	1.00(reference)	1.21(0.85–1.72)	1.63(1.16–2.30)	2.25(1.58–3.23)	<.0001
OR^b^	1.00(reference)	1.19(0.83–1.69)	1.59(1.12–2.25)	2.28(1.59–3.29)	<.0001
OR^c^	1.00(reference)	1.18(0.83–1.68)	1.57(1.11–2.23)	2.28(1.58–3.29)	<.0001
**NHS II**					
Quartile values	≤19.6	19.6–25.5	25.5–31.4	>31.4	
n, case/control	24/604	26/602	34/603	39/606	
OR^a^	1.00(reference)	1.19(0.67–2.10)	1.62(0.93–2.81)	1.93(1.12–3.35)	0.01
OR^b^	1.00(reference)	1.20(0.68–2.13)	1.64(0.94–2.86)	1.94(1.11–3.38)	0.01
OR^c^	1.00(reference)	1.20(0.67–2.13)	1.63(0.93–2.85)	1.93(1.10–3.37)	0.01
**Total**					
n, case/control	93/1010	112/1022	142/1008	163/1016	
OR^d^	1.00(reference)	1.19(0.88–1.61)	1.59(1.19–2.13)	2.07(1.54–2.79)	<.0001
OR^e^	1.00(reference)	1.17(0.87–1.59)	1.55(1.16–2.08)	2.07(1.53–2.80)	<.0001
OR^f^	1.00(reference)	1.17(0.86–1.58)	1.54(1.15–2.07)	2.07(1.52–2.80)	<.0001

Abbreviation: OR, odds ratio.

a Adjusted for age at blood draw, season of blood draw, lab batch.

b Adjusted for age at blood draw, season of blood draw, lab batch, hair color, burning tendency, numbers of sunburns.

c Adjusted for age at blood draw, season of blood draw, lab batch, hair color, burning tendency, numbers of sunburns, UVB flux.

d Adjusted for age at blood draw, season of blood draw, lab batch, cohort.

e Adjusted for age at blood draw, season of blood draw, lab batch, cohort, hair color, burning tendency, numbers of sunburns.

f Adjusted for age at blood draw, season of blood draw, lab batch, cohort, hair color, burning tendency, numbers of sunburns, UVB flux.

**Table 3:** Odds ratios of SCC by quartile of plasma vitamin D concentrations in NHS and NHS II.

	25-Hydroxyvitamin D concentrations, ng/mL				P for trend
	1st quartile	2nd quartile	3rd quartile	4th quartile	
**NHS**					
Quartile values	≤20.4	20.4–27.0	27.0–34.2	>34.2	
n, case/control	9/406	12/420	22/405	24/410	
OR^a^	1.00(reference)	1.43(0.59–3.47)	2.84(1.27–6.37)	3.56(1.56–8.13)	0.0006
OR^b^	1.00(reference)	1.48(0.60–3.60)	2.95(1.30–6.70)	3.79(1.63–8.80)	0.0004
OR^c^	1.00(reference)	1.49(0.61–3.66)	3.04(1.33–6.95)	3.96(1.68–9.34)	0.0004
**NHS II**					
Quartile values	≤19.6	19.6–25.5	25.5–31.4	>31.4	
n, case/control	1/604	1/602	2/603	4/606	
OR^a^	1.00(reference)	1.03(0.06–16.79)	1.94(0.16–22.76)	3.43(0.34–34.42)	0.20
OR^b^	1.00(reference)	1.33(0.08–22.46)	2.54(0.20–32.28)	4.22(0.39–45.28)	0.17
OR^c^	1.00(reference)	1.48(0.08–27.86)	2.62(0.19–36.30)	4.95(0.41–59.28)	0.15
**Total**					
n, case/control	10/1010	13/1022	24/1008	28/1016	
OR^d^	1.00(reference)	1.37(0.59–3.18)	2.73(1.27–5.87)	3.62(1.67–7.84)	0.0002
OR^e^	1.00(reference)	1.43(0.61–3.33)	2.87(1.32–6.22)	3.87(1.76–8.49)	0.0001
OR^f^	1.00(reference)	1.40(0.60–3.28)	2.81(1.29–6.12)	3.77(1.70–8.36)	0.0002

a Adjusted for age at blood draw, season of blood draw, lab batch.

b Adjusted for age at blood draw, season of blood draw, lab batch, hair color, burning tendency, numbers of sunburns.

c Adjusted for age at blood draw, season of blood draw, lab batch, hair color, burning tendency, numbers of sunburns, UVB flux.

d Adjusted for age at blood draw, season of blood draw, lab batch, cohort.

e Adjusted for age at blood draw, season of blood draw, lab batch, cohort, hair color, burning tendency, numbers of sunburns.

f Adjusted for age at blood draw, season of blood draw, lab batch, cohort, hair color, burning tendency, numbers of sunburns, UVB flux.

A previous study indicated that seasonal variation may introduce biased results of 25(OH)D levels [28]. Therefore, we conducted stratified analysis to test the interaction of vitamin D and season of blood draw. We modeled season of blood draw as a three-category variable (summer, spring and fall, winter). We tested two multiplicative interaction terms by the likelihood ratio test, comparing the model with the interaction terms with the model containing just the main effects of vitamin D and season of blood draw variables, along with the same covariates. To test interaction of vitamin D and UVB flux, we modeled UVB flux as a dichotomous variable (113 as a cutoff point). We tested one multiplicative interaction term by the likelihood ratio test. Finally, to summarize multiple variables, we constructed a multivariate confounder score [29] to create a pigmentation score for BCC. Briefly, we applied the logistic regression coefficients from a multivariate model including age, hair color, burning tendency, and the number of sunburns of BCC to each individual’s values for the latter three of these variables and summed the values to compute a pigmentation score. We used this score to identify participants with light and dark pigmentation phenotypes based on the median score.

We tested one multiplicative interaction term between vitamin D and pigmentation by the likelihood ratio test. Statistical analyses were conducted using SAS software (version 9, SAS Institute, Cary, NC). All statistical tests were two-sided.

**Table 4:** Odds ratios of BCC by quartile of plasma vitamin D concentrations in NHS and NHS II (stratified by season of blood draw).

season of blood draw	25-Hydroxyvitamin D concentrations, ng/mL^d^				P for trend
	1st quartile	2nd quartile	3rd quartile	4th quartile	
**Summer**					
n, case/control	25/138	33/224	44/259	56/333	
OR^a^	1.00(reference)	0.73(0.40–1.34)	0.90(0.50–1.64)	0.95(0.53–1.72)	0.81
OR^b^	1.00(reference)	0.68(0.37–1.27)	0.88(0.48–1.62)	0.93(0.50–1.70)	0.82
OR^c^	1.00(reference)	0.68(0.37–1.27)	0.88(0.48–1.62)	0.93(0.51–1.71)	0.81
**Spring and fall**					
n, case/control	41/536	61/550	84/517	95/516	
OR^a^	1.00(reference)	1.46(0.93–2.29)	2.26(1.46–3.49)	3.08(1.99–4.78)	<.0001
OR^b^	1.00(reference)	1.45(0.92–2.29)	2.11(1.36–3.29)	2.97(1.90–4.63)	<.0001
OR^c^	1.00(reference)	1.45(0.92–2.29)	2.10(1.35–3.28)	2.97(1.90–4.63)	<.0001
**Winter**					
n, case/control	37/331	31/243	38/229	39/162	
OR^a^	1.00(reference)	1.38(0.79–2.40)	1.41(0.81–2.45)	2.29(1.27–4.13)	0.01
OR^b^	1.00(reference)	1.35(0.76–2.40)	1.47(0.83–2.60)	2.58(1.39–4.77)	0.005
OR^c^	1.00(reference)	1.33(0.75–2.37)	1.44(0.81–2.57)	2.53(1.36–4.72)	0.006

a Adjusted for age at blood draw, lab batch, cohort.

b Adjusted for age at blood draw, lab batch, cohort, hair color, burning tendency, numbers of sunburns.

c Adjusted for age at blood draw, lab batch, cohort, hair color, burning tendency, numbers of sunburns, UVB flux.

d P for interaction between season of blood draw and 25-hydroxyvitamin D is 0.09 after adjusted for age at blood draw, lab batch, cohort.

**Table 5:** Odds ratios of BCC by quartile of plasma vitamin D concentrations in NHS and NHS II (stratified by UVB flux).

UVB flux	25-Hydroxyvitamin D concentrations, ng/mL^c^				P for trend
	1st quartile	2nd quartile	3rd quartile	4th quartile	
**≤113**					
n, case/control	60/666	88/648	94/564	113/556	
OR^a^	1.00(reference)	1.49(1.02–2.17)	1.82(1.24–2.67)	2.62(1.77–3.89)	<.0001
OR^b^	1.00(reference)	1.49(1.02–2.18)	1.81(1.23–2.67)	2.66(1.78–3.97)	<.0001
**>113**					
n, case/control	43/343	37/374	72/443	78/458	
OR^a^	1.00(reference)	0.79(0.47–1.31)	1.24(0.79–1.95)	1.45(0.90–2.33)	0.04
OR^b^	1.00(reference)	0.80(0.47–1.34)	1.25(0.78–1.98)	1.52(0.94–2.46)	0.03

a Adjusted for age at blood draw, season of blood draw, lab batch, cohort.

b Adjusted for age at blood draw, season of blood draw, lab batch, cohort, hair color, burning tendency, numbers of sunburns.

c P for interaction between UVB flux and 25-hydroxyvitamin D is 0.07 after adjusted for age at blood draw, season of blood draw, lab batch, cohort.

**Table 6:** Odds ratios of BCC by quartile of plasma vitamin D concentrations in NHS and NHS II (stratified by pigmentation).

pigmentation	25-Hydroxyvitamin D concentrations, ng/mL^c^				P for trend
	1st quartile	2nd quartile	3rd quartile	4th quartile	
**Light pigmentation**					
n, case/control	46/522	53/531	81/488	97/498	
OR^a^	1.00(reference)	1.08(0.69–1.69)	1.84(1.20–2.81)	2.35(1.53–3.61)	<.0001
OR^b^	1.00(reference)	1.08(0.69–1.68)	1.81(1.18–2.78)	2.31(1.50–3.56)	<.0001
**Dark pigmentation**					
n, case/control	57/488	72/491	85/520	94/518	
OR^a^	1.00(reference)	1.26(0.84–1.91)	1.38(0.92–2.08)	1.84(1.20–2.82)	0.005
OR^b^	1.00(reference)	1.26(0.83–1.91)	1.38(0.91–2.08)	1.85(1.21–2.85)	0.005

a Adjusted for age at blood draw, season of blood draw, lab batch, cohort.

b Adjusted for age at blood draw, season of blood draw, lab batch, cohort, UVB flux.

c P for interaction between UVB flux and 25-hydroxyvitamin D is 0.15 after adjusted for age at blood draw, season of blood draw, lab batch, cohort.

## Results

The characteristics of women by quartiles of plasma 25(OH)D concentration in two cohorts are presented in Table 1. Women who provided blood samples in summer tended to have higher 25(OH)D levels than those whose blood was drawn in winter, which is consistent with previously published data [30]. In addition, women with higher 25(OH)D levels were more likely to reside in areas of higher UVB flux.

The associations between plasma vitamin D levels and BCC and SCC were examined separately (Tables 2 and 3). Similar significantly positive findings were obtained between plasma 25(OH)D and risk of BCC in both cohorts in multivariate models (P for trend <0.0001 and  = 0.01). Women in the highest quartile of 25(OH)D had about 2-fold increased risk of BCC compared with women in the lowest quartile, both in NHS (OR = 2.28, 95% CI = 1.58–3.29) and in NHS II (OR = 1.93, 95% CI = 1.10–3.37). Results were similar when combining the data of NHS and NHS II (OR = 2.07, 95% CI = 1.52–2.80, P for trend <0.0001) (Table 2).

We noted significantly positive associations between quartiles of 25(OH)D levels and SCC in NHS (OR = 3.96, 95% CI = 1.68–9.34, P for trend  = 0.0004). In NHS II, although a similar trend was observed, the result was not statistically significant because only eight cases were identified. Overall, the positive association between 25(OH)D levels and SCC was significant after combining the two cohorts (P for trend  = 0.0002). Women in the highest quartile of 25(OH)D had more than a 3-fold increased risk for SCC compared with women in the lowest quartile (OR = 3.77, 95% CI = 1.70–8.36) (Table 3).

We further examined the association between plasma 25(OH)D levels and BCC risk stratified by season of blood draw, UVB flux, and pigmentation (Tables 4, 5, 6). There was a significant positive association between 25(OH)D and risk of BCC among women with blood collection in spring/fall (OR = 2.97, 95% CI = 1.90–4.63) or in winter (OR = 2.53, 95% CI = 1.36–4.72), whereas no association was observed among women with blood collection in summer (OR = 0.93, 95% CI = 0.51–1.71) (P for interaction  =  0.09) (Table 4). In the stratified analysis by UVB flux, women who lived in lower UVB flux (≤113) areas tended to have higher OR (2.66, 95% CI =  1.78–3.97) when comparing the top to bottom quartile of 25(OH)D levels (P for interaction = 0.07) (Table 5). The positive association between 25(OH)D and risk of BCC appeared to be stronger among women with light pigmentation (P for interaction = 0.15) (Table 6).

## Discussion

In this study, higher plasma 25(OH)D levels were associated with greater non-melanoma skin cancer risk among women from two large cohorts. In subgroup analysis, higher plasma 25(OH)D tended to increase risk of BCC in women whose blood was collected outside the summer season, who were from areas with less UVB flux (≤113), and among those with light pigmentation. To our knowledge, the current study is one of the largest to provide important evidence on the association of pre-diagnostic plasma 25(OH)D levels with non-melanoma skin cancer.

Vitamin D is predominantly produced in the skin by UVB exposure, which usually contributes 80–90% to total vitamin D in the human body [12]. Our data suggest that one-time measurement of plasma vitamin D levels may reasonably reflect long-term sun exposure. The validity of single 25(OH)D measures was also evaluated in NHS. The intraclass correlation coefficients for plasma 25(OH)D measured over 3 years and over 10–11 years were 0.72 and 0.50, respectively [31]. This indicates that a single 25(OH)D measurement is fairly reproducible over years and reasonably reflects long-term vitamin D status. It is well known that sun exposure is the main cause of skin cancers. Therefore, plasma 25(OH)D levels may predict the risk of non-melanoma skin cancer.

Recently, Eide et al.[10] reported a positive relationship between plasma levels of 25(OH)D and non-melanoma skin cancer (adjusted OR, 1.8; 95% CI, 1.1–2.9), including SCC and BCC, in a study of 3223 white health maintenance organization patients who sought advice about the risk of osteoporosis or low bone density. The 25(OH)D levels were similarly positively associated with non-melanoma skin cancer risk at anatomical locations less exposed to UV (adjusted OR, 2.2; 95% CI, 0.7–7.0). In another nested case-control study, Asgari et al. [11] also found an increased risk of BCC with higher vitamin D levels among 220 BCC patients and 220 matched controls from the Kaiser Permanente Northern California Health Maintenance Organization (adjusted OR, 2.09; 95% CI, 0.95–4,58). The results of this study support the findings from Eide et al. and Asgari et al., with similar risk estimated for BCC. However, Tang et al. [9] found an inverse association between higher plasma 25(OH)D levels and risk of non-melanoma skin cancer (OR, 0.6; 95% CI, 0.37–0.98) among 930 white men from the Osteoporotic Fractures in Men Study. It should be noted that all the participants in this study and most of the patients of Eide et al. were women, but the participants in study of Tang et al. were men. Furthermore, the cohorts in our study were followed up for more than 10 years, and the Eide et al. cohort was followed for almost 10 years, but the patients of Tang et al. were followed up for only 5 years. Lastly, our study had the largest sample size. These differences may explain the variations between the results in this study and those of Tang et al.

Stratified analysis on season suggests that 25(OH)D is positively associated with BCC risk among women whose blood was collected outside the summer months. In addition, we observed that the associations between plasma 25(OH)D and BCC risk were stronger among women from areas with less UVB flux. Similar to previous report [32], our data support that plasma 25(OH)D could reasonably reflect long-term vitamin D status more accurately outside the summer season or in areas of less UVB flux. In a case-control study, an inverse association between 25(OH)D and lung cancer was also found only among those whose blood was drawn during the darker months [33].

Vitamin D may be of importance in skin cancer development. Although the data from various types of cancer suggest a benefit from high vitamin D levels, they could be a marker of high sun exposure and an increased risk of skin cancer. There is *in vitro* evidence that vitamin D treatment decreases cell growth and metastasis [15], [16], but most of the cases studied were melanoma. Evidence *in vitro* for the role of vitamin D in non-melanoma skin cancer is still limited. Mice with inactivated vitamin D receptor had more non-melanoma skin cancer [34]. Vitamin D3 inhibited the proliferation of basal cell carcinomas by inhibiting the hedgehog signaling pathway [16]. The evidence in humans of a positive relationship between vitamin D and non-melanoma skin cancer suggests that UV exposure may have a predominant adverse influence that exceeds any putative benefit from the higher levels of vitamin D.

The strengths of the current study include its prospective design, large well-characterized study population, long follow-up duration, and data on potential confounders. The limitation is that blood samples were not assayed at the same time and in the same laboratory, although we have controlled for batch variation in the multivariate models.

In conclusion, this prospective study found a positive association between plasma 25(OH)D levels and risk of non-melanoma skin cancer. Additionally, for BCC, this association was more apparent among women with blood collection outside the summer season, women from areas with less UVB flux, and light-pigmented women. Considering that most circulating vitamin D is due to sun exposure, the positive association between plasma vitamin D and non-melanoma skin cancer is confounded by sun exposure. Our results suggest that one-time measurement of plasma vitamin D may reasonably reflect long-term sun exposure and predict the risk of non-melanoma skin cancer.

## Acknowledgments

We thank the participants in the NHS and NHS II Studies for their dedication and commitment. We also thank the following state cancer registries for their help: AL, AZ, AR, CA, CO, CT, DE, FL, GA, ID, IL, IN, IA, KY, LA, ME, MD, MA, MI, NE, NH, NJ, NY, NC, ND, OH, OK, OR, PA, RI, SC, TN, TX, VA, WA, WY.
